# Calpain‐4 Knockdown Modulates Cholesterol Metabolism and LXRα Nuclear Localization in Experimental Alcohol‐Related Liver Disease

**DOI:** 10.1111/acer.70356

**Published:** 2026-06-17

**Authors:** Noriko Kitano, Jiang Li, Sam Taborski, Charis‐Marie Vanderpuye, Pooja Muddasani, Sudrishti Chaudhary, Jia‐Jun Liu, Silvia Liu, Juliane I. Beier, Josepmaria Argemi, Ramon Bataller, Gavin E. Arteel

**Affiliations:** ^1^ Division of Gastroenterology, Hepatology and Nutrition, Department of Medicine University of Pittsburgh Pittsburgh Pennsylvania USA; ^2^ Pharmacology and Chemical Biology University of Pittsburgh Pittsburgh Pennsylvania USA; ^3^ Organ Pathobiology and Therapeutics Institute University of Pittsburgh Pittsburgh Pennsylvania USA; ^4^ Pittsburgh Liver Research Center University of Pittsburgh Pittsburgh Pennsylvania USA; ^5^ Department of Environmental and Occupational Health University of Pittsburgh Pittsburgh Pennsylvania USA; ^6^ Liver Unit, Department of Internal Medicine Clinical University of Navarra Pamplona Navarra Spain; ^7^ Institut d'Investigacions Biomediques August Pi i Sunyer (IDIBAPS) University of Barcelona Barcelona Spain

**Keywords:** alcohol‐related liver disease, calpain, HMGCR, lipid metabolism, LXRα

## Abstract

**Background:**

Ethanol affects lipid metabolism through multiple pathways, leading to fatty liver development in most alcohol‐related liver disease (ALD) patients. Recent studies have highlighted the role of calpain, a calcium‐dependent protease, in liver inflammation and fibrosis. Calpain activity is regulated by its essential subunit, Capns1 (calpain‐4), which stabilizes and modulates the activity of its catalytic isoforms, calpain‐1 and calpain‐2. This study investigated calpain's impact on lipid metabolism in ALD.

**Methods:**

Six‐week‐old C57Bl6/J mice were injected with rAAV8 vectors encoding Capns1 shRNA or control vectors. After 4 weeks, mice underwent a 10‐day period of ad libitum ethanol consumption, followed by a single gavaged ethanol administration on day 11.

**Results:**

Capns1 knockdown attenuated ethanol‐induced microvesicular steatosis. Hepatic triglyceride and free fatty acid levels were not significantly altered, whereas cholesterol levels were significantly reduced in the ethanol group with Capns1 knockdown. *Cpt1a* expression increased significantly in the ethanol group with Capns1 knockdown. Western blot analysis revealed increased Cleaved‐3‐hydroxy‐3‐methyl‐glutaryl‐coenzyme A reductase (HMGCR) to Pro‐HMGCR ratio in Capns1‐knockdown mice, suggesting reduced HMGCR activity and suppressed cholesterol biosynthesis. LXRα expression was mainly increased in the cytoplasm in the ethanol group, and following Capns1 knockdown, it was relocalized to the nucleus via its activation. In addition, RNA sequencing analysis indicated that Capns1 knockdown contributes to the reprogramming of ethanol‐induced disruptions in metabolic pathways, primarily those involving cholesterol metabolism.

**Conclusion:**

Further investigation into the relationship between Capns1 and cholesterol biosynthesis proteins may provide insights into using calpain inhibitors as a therapeutic approach for alcohol‐related liver disease.

## Graphical abstract

Calpain‐4 knockdown in alcohol‐related liver disease promotes LXRα nuclear translocation and HMGCR cleavage, enhancing cholesterol efflux via upregulation of ABCA1/ABCG1 and CPT‐1. This mechanism reduces hepatic cholesterol accumulation and microvesicular steatosis without affecting triglycerides or inflammatory markers, identifying calpain‐4 as a novel regulator of cholesterol metabolism in ALD.

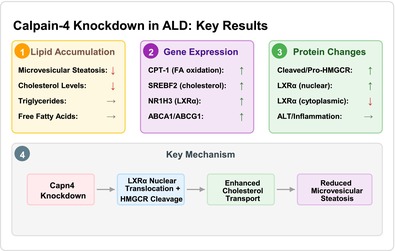

**Abbreviations**
ABCA1: ATP‐binding cassette transporter A1
ABCG1: ATP‐binding cassette transporter G1
ALD: alcohol‐related liver disease
ALT: alanine aminotransferase
ASH: alcohol‐related steatohepatitis
AST: aspartate aminotransferase
AUROC: area under the receiver operating characteristic curve
BSA: bovine serum albumin
Capn1: calpain‐1
Capn2: calpain‐2
Capns1: calpain small subunit 1
CD68: cluster of differentiation 68
Cpt1a: carnitine palmitoyltransferase 1A
Cyp7a1: cytochrome P450 family 7 subfamily A member 1
CysPC: calpain cysteine protease core
Dgat2: diacylglycerol O‐acyltransferase 2
E11.5: embryonic day 11.5
EIF2AK4: eukaryotic translation initiation factor 2 alpha kinase 4
ER: endoplasmic reticulum
F4/80: EGF‐like module‐containing mucin‐like hormone receptor‐like 1
Fasn: fatty acid synthase
GCN2: general control nonderepressible 2
HMGCR: 3‐hydroxy‐3‐methylglutaryl‐CoA reductase
Lcat: lecithin–cholesterol acyltransferase
LDLR: low‐density lipoprotein receptor
LXRα: liver X receptor alpha
Ly6g: lymphocyte antigen 6 complex locus G6D
MLXIPL: MLX interacting protein‐like
Pai‐1: plasminogen activator inhibitor‐1
ROS: reactive oxygen species
RT‐PCR: reverse transcription polymerase chain reaction
SDS: sodium dodecyl sulfate
Srebf2: sterol regulatory element‐binding transcription factor 2
SREBP2: sterol regulatory element‐binding protein 2
Tnfα: tumor necrosis factor alpha

## 1 Introduction

Excessive alcohol consumption is a significant global health concern, affecting millions of individuals worldwide. Chronic ethanol consumption is well known to cause dysregulation of lipid metabolism. Following heavy alcohol consumption (> 60 g ethanol/day for ≥ 2 weeks), ~90% of individuals develop hepatic steatosis, of whom 20%–40% may develop fibrosis and 10%–35% may develop steatohepatitis (Staufer and Stauber 2023). Despite the high prevalence of alcohol‐related liver disease (ALD), effective therapeutic strategies remain elusive.

Although fatty liver is often considered a benign stage of liver disease, its severity strongly correlates with progression to advanced ALD (Parker et al. 2018; Seitz et al. 2018). Early stages of ALD, including fatty liver, are typically asymptomatic and can be reversed with abstinence (Stickel et al. 2003). However, due to the absence of clinical symptoms or significant abnormalities blood markers of liver damage (e.g., aminotransferases), the severity of early‐stage ALD is often underestimated. These observations suggest that early metabolic alterations may influence disease susceptibility and progression, highlighting the importance of understanding regulatory mechanisms governing lipid and cholesterol homeostasis during the initial stages of ALD (You and Arteel 2019).

Calpain‐1 (μ‐calpain) and calpain‐2 (m‐calpain) are calcium‐dependent cysteine proteases that function as heterodimers consisting of distinct 80 kDa catalytic subunits encoded by Capn1 and Capn2, respectively, and a common 28 kDa regulatory subunit encoded by Capns1 (also known as Capn4) (Goll et al. 2003). The regulatory subunit is essential for maintaining the stability and activity of both catalytic subunits, as genetic ablation of Capns1 results in rapid degradation of Capn1 and Capn2 proteins despite continued Capn1 and Capn2 mRNA expression (Arthur et al. 2000). However, when the regulatory subunit is present, increases in the catalytic subunits directly enhance calpain proteolytic activity. Studies in vascular smooth muscle cells and cardiomyocytes have demonstrated that overexpression of Capn1 increases substrate cleavage and downstream cellular responses (Jiang et al. 2008; Ni et al. 2016), suggesting that the expression level of the catalytic subunits can influence calpain activity under physiological conditions when Capns1 function is intact. These observations suggest that modulation of Capn1 and Capn2 expressions may represent an important regulatory mechanism for controlling calpain‐mediated proteolysis in diverse pathological contexts. Recent studies have highlighted the role of calpain in liver inflammation and fibrosis (Li et al. 2023; Sato et al. 2023). Nevertheless, its specific role in ALD remains poorly understood. This study examined the involvement of Capns1 in ALD pathogenesis using a chronic alcohol‐induced liver injury model, with particular focus on the relationships between Capns1, inflammation, and lipid metabolism.

## 2 Materials and Methods

Please see Supporting Information for additional details on Methods.

### 2.1 Publicly Available Liver RNA Sequencing Analysis

RNA‐seq data were obtained from normal livers (*n* = 10) and from biopsies of patients with early silent ALD (ASH, *n* = 11), non‐severe alcohol‐related steatohepatitis (AH) (*n* = 9), and severe AH (*n* = 9) from the InTEAM Consortium– Alcohol‐related Hepatitis Liver RNA‐Seq study, sponsored by the National Institute of Alcohol Abuse and Alcoholism (NIAAA, USA). The study details and sequencing data can be found in the Database of Genotypes and Phenotypes (dbGAP, phs001807.v1. p1) of the National Institutes of Health (NIH, USA). The basic clinical and laboratory data of the patients included in this study, the methods used to extract RNA and perform deep RNA‐seq, and the bioinformatic pipelines used to determine transcript counts have been described previously (Argemi et al. 2019).

### 2.2 Animals and Treatments

**FIGURE 1:**
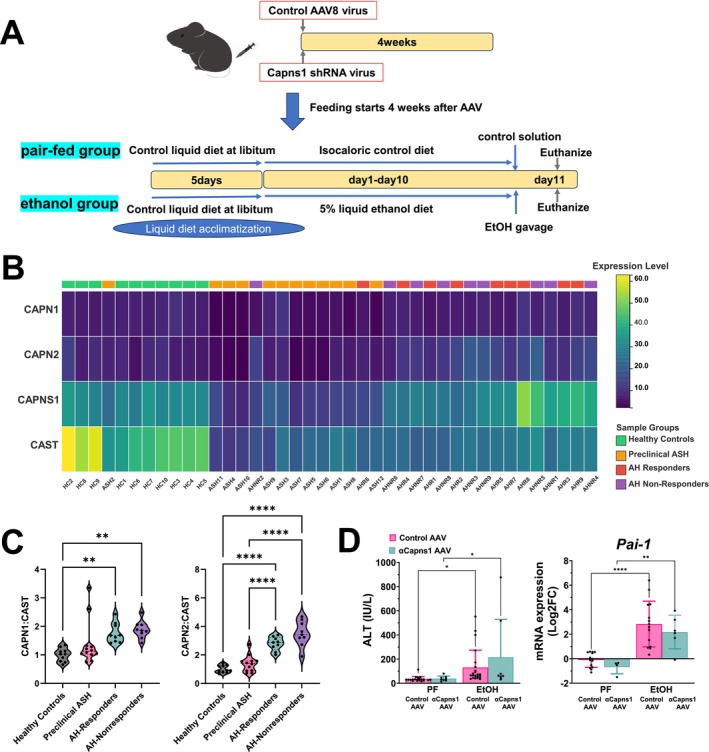
Changes in calpain family expression in human alcohol‐related liver disease and effects of ethanol exposure and Capns1 knockdown in a mouse model. Panel (A) The schedule of the study is depicted, illustrating the protocol for ethanol administration. Mice are initially fed the control diet ad libitum for 5 days to acclimatize them to the liquid diet. Afterward, the ethanol group is allowed free access to the ethanol diet containing 5% (vol/vol) ethanol for 10 days, and the control group is pair‐fed an isocaloric control diet. On day 11, the ethanol‐fed and pair‐fed mice are gavaged with a single dose of ethanol (5 g kg^−1^ body weight) or isocaloric maltose dextrin, respectively, and euthanized 9 h later. Panel (B) RNA‐seq analysis of human liver samples from healthy controls (HC), preclinical ASH (ASH), AH responders (AHR), and AH nonresponders (AHNR) obtained from the publicly available dbGAP dataset (phs001807.v1.p1). This heatmap shows normalized expression levels of *CAPN1, CAPN2, CAST*, and *CAPNS1* genes in each group. Panel (C) Ratios of *CAPN1/CAST* and *CAPN2/CAST* were calculated from the same RNA‐seq dataset as an indirect index of calpain activity. Bar graphs show the mean ± SEM of these ratios in HC, ASH, AHR, and AHNR groups. Panel (D) To assess liver injury, we measured serum ALT level, the expression of proinflammatory genes (*Pai‐1*). *p < 0.05, **p < 0.01, and ****p < 0.0001. AAV, adeno‐associated virus; EtOH, ethanol‐fed; PF, pair‐fed.

Six‐week‐old, male C57Bl/6J mice purchased from Jackson Laboratory (Bar Harbor, ME) were housed in a pathogen‐free barrier facility accredited by the Association for Assessment and Accreditation of Laboratory Animal Care, and procedures were approved by the local Institutional Animal Care and Use Committee. Animals were allowed standard laboratory chow and water ad libitum. For Capns1‐knockdown study, following a 5‐day acclimation period, the mice were divided into two groups. The first group received a 10‐day ad libitum diet of 5% liquid ethanol, with subgroups receiving either control AAV8 virus or Capns1 shRNA virus injections for 4 weeks before diet consumption (Figure 1A). The second control group received an isocaloric control diet with the same subgroups. On day 11, mice were orally gavaged with ethanol (5 g/kg body weight) or control solution. All procedures related to the feeding protocol were performed according to the chronic and binge ethanol feeding model described by Bertola et al. (2013). After 9 h of ethanol administration, the mice were anesthetized with ketamine/xylazine (100/15 mg/kg, i.p.). Blood was collected from the vena cava just prior to sacrifice by exsanguination and citrated plasma was stored at −80°C for further analysis. Portions of liver tissue were frozen immediately in liquid nitrogen, while others were fixed in 10% neutral buffered formalin or embedded in frozen specimen medium (Tissue‐Tek OCT compound, Sakura Finetek, Torrance, CA) for subsequent sectioning and mounting on microscope slides. The body weight of the mice and the liver weight at the time of sacrifice were measured.

### 2.3 Biochemical Assays and Histology

Plasma levels of aspartate transaminase (AST) and alanine aminotransferase (ALT) were determined spectrophotometrically using standard kits (Thermo Fisher Scientific, Waltham, MA). Formalin‐fixed, paraffin‐embedded sections were cut at 5 μm and mounted on glass slides. Deparaffinized sections stained with hematoxylin and eosin (H&E) and pathology were assessed in a blinded manner. Hepatic lipids were determined using standard clinical chemistry reagents for cholesterol (CL) and triglycerides (TG) (Infinity, Thermo Fisher Scientific, Waltham, MA), for free fatty acids (FFA) (Cell Biolabs Inc., San Diego, CA), and β‐hydroxybutyrate (βHB) (Colorimetric, Cell Biolabs Inc., San Diego, CA) (See Table S1). From H&E‐stained sections, lipid accumulation was quantified by steatosis scoring, where a score of 0 indicates 0% tissue coverage by lipid droplets (LDs), 1 = 1%–25% coverage, 2 = 26%–50% coverage, 3 = 51%–75% coverage, and 4 = 76%–100% coverage. For detection of hepatic Capns1 and Liver X Receptor Alpha (LXRα), paraffin‐embedded liver sections were incubated with biotinylated antibodies against Capns1 and LXRα (DAKO, Carpenteria, CA). The sections were counterstained with hematoxylin (Sigma, St. Louis, MO), and immunoreactivity was visualized using a DAB detection kit (DAKO, Carpenteria, CA). All staining procedures were performed using the Vectastain kit (Torrance, CA) according to the manufacturer's instructions. Images were captured using Metamorph software (Molecular Devices, Sunnyvale, CA).

### 2.4 RNA Isolation and Real‐Time Reverse Transcription Polymerase Chain Reaction (Real‐Time RT‐PCR)

RNA was extracted immediately following sacrifice from fresh liver samples using RNA Stat60 (Tel‐Test, Ambion, Austin, TX) and chloroform. RNA concentrations were determined spectrophotometrically and 1 μg of total RNA was reverse transcribed using the QuantiTect Reverse Transcription Kit (Qiagen, Valencia, CA). Real‐time reverse transcription polymerase chain reaction (Real‐time RT‐PCR) was performed using a StepOne real‐time PCR system (Thermo Fisher Scientific, Grand Island, NY) using the Taqman Universal PCR Master Mix (Life Technologies, Carlsbad, CA). Primers and probes were ordered as commercially available kits (Thermo Fisher Scientific, Grand Island, NY; see Table S2). The comparative C_T_ method was used to determine fold differences between the target genes and an endogenous reference gene (18S). Results were reported as Log2FC of naïve control (2^−ΔΔ*C*T^).

### 2.5 RNA Sequencing and Differential Expression Analysis

To investigate the transcriptomic alterations induced by ethanol administration and their modulation by Capns1 knockdown, we performed RNA‐seq followed by differential expression analysis. Total RNA was extracted from liver tissues of four experimental groups: (1) pair‐fed wild‐type (defined as Control), (2) ethanol‐fed wild‐type (defined as EtOH), (3) pair‐fed Capns1 knockdown (defined as Capns1 KD), and (4) ethanol‐fed Capns1 knockdown (defined as EtOH+Capns1 KD) mice.

The bioinformatic pipelines used to determine transcript counts have been described previously (Argemi et al. 2019). Differentially expressed genes (DEGs) were identified using DESeq2 with *p* < 0.05 and |log_2_(fold change)| > 1. Pathway analyses were conducted using Ingenuity Pathway Analysis (IPA) software from Qiagen in Valencia, CA (http://www.ingenuity.com). This software was utilized for canonical pathway analysis and network discovery. IPA was used to identify top canonical and enriched biological pathways based on DEGs. The Venn diagram was generated using the online tool *Venny* (https://bioinfogp.cnb.csic.es/tools/venny/) and *Venn Diagram Plotter* (Pacific Northwest National Laboratory) to visualize the overlap between DEGs in these comparisons.

### 2.6 Immunoblots

Liver samples were homogenized in lysis buffer (20 mM Tris/Cl, pH 7.5, 150 mM NaCl, 1 mM EDTA, 1 mM EGTA, 1% (w/v) Triton X‐100), containing protease and phosphatase inhibitor cocktails (Sigma, St. Louis, MO). Samples were loaded onto Invitrogen Bolt 4%–12% Bis‐Tris Plus gels (Thermo Fisher Scientific, Waltham, MA, USA) and subjected to electrophoresis. Gels were then blotted onto polyvinylidene difluoride membranes using the iBlot 2 PVDF Mini Stacks (Thermo Fisher Scientific, Waltham, MA, USA). The membranes were washed in TBST buffer and blocked with TBST containing 5% bovine serum albumin. Primary antibodies against HMGCR, ABCA1, ABCG1, and VCP. LXRα and GAPDH (see Table S3) were used. After incubation with HRP‐conjugated secondary antibodies, protein bands were visualized using an Enhanced Chemiluminescence kit (Pierce, Rockford, IL) and Hyperfilm (GE Healthcare, Piscataway, NJ). The results were visualized and densitometric analysis was performed using an iBright imager and onboard software from Invitrogen (Waltham, MA).

### 2.7 Statistical Analysis

Quantitative data are reported means ± SEM. Statistical analyses were performed using two‐way ANOVA followed by Fisher's least significant difference (LSD) test. A significance threshold of *p* < 0.05 was predetermined. Asterisks indicate statistical significance (*p < 0.05, **p < 0.01, ***p < 0.001, and ****p < 0.0001).

## 3 Results

### 3.1 Changes in Calpain Family Expression in Human Alcohol‐Related Liver Disease and Effects of Ethanol Exposure and Capns1 Knockdown in a Mouse Model

Calpains are calcium‐dependent cysteine proteases characterized by the calpain cysteine protease core motif in their genes (Goll et al. 2003). These enzymes regulate crucial biological processes, including cell migration, apoptosis, and synaptic plasticity through targeted protein cleavage (Goll et al. 2003; Ono and Sorimachi 2012). The two primary isoforms, Capn1 and Capn2, are activated by calcium signaling (Goll et al. 2003; Ono and Sorimachi 2012). Their activity is specifically regulated by calpastatin (Cast), an endogenous inhibitor that binds and colocalizes with calpain proteases (Miyazaki et al. 2015). Capns1 functions as a small regulatory subunit that promotes and stabilizes the expression of both Capn1 and Capn2 (Bai et al. 2009; Jang et al. 2024; Zhang et al. 2013).

To further explore the potential role of the calpain family in ALD, we performed a secondary analysis using a publicly available dataset from the Database of Genotypes and Phenotypes (dbGAP, phs001807.v1.p1; National Institutes of Health, USA). RNA‐seq of human liver samples from healthy controls (HC), preclinical ASH (ASH), AH responder (AHR), and AH non‐responder (AHNR) revealed altered calpain family gene expression (Figure 1B). Among these alterations, *CAPN2* expression was increased in clinical ALD (AHR and AHNR) subgroups compared with healthy controls, whereas *CAST* was markedly decreased. Since *CAST* is the key inhibitor of calpain activity, the *CAPN* to *CAST* ratio is an indirect index of calpain activity (Shields et al. 1999). The ratio of *CAPN1* or *CAPN2* to *CAST* was significantly elevated in both AHR and AHNR groups (Figure 1C), suggesting increased calpain activity without a corresponding increase in its endogenous inhibitor *CAST* in advanced ALD.

To examine the functional significance of these observations, we established a chronic ethanol‐feeding mouse model in which Capns1 was knocked down via AAV (Figure 1A). As previously observed, ethanol exposure via this regimen increased serum ALT levels (Ki et al. 2010) and Pai‐1 expression (Warner et al. 2021). Previous studies have shown that Pai‐1 is involved in the development and progression of alcohol‐related liver disease (Arteel 2008). These changes caused by ethanol were not significantly attenuated by Capns1 knockdown (Figure 1D), suggesting that Capns1 knockdown does not markedly affect acute inflammatory signaling in this model, although macrophage‐associated markers showed modest changes (Figure S1). In addition, other markers and genes associated with liver injury were also evaluated (see Figure S1).

### 3.2 Changes in Calpain Family Expression Induced by Ethanol Exposure: Effect of Capns1 Knockdown

**FIGURE 2:**
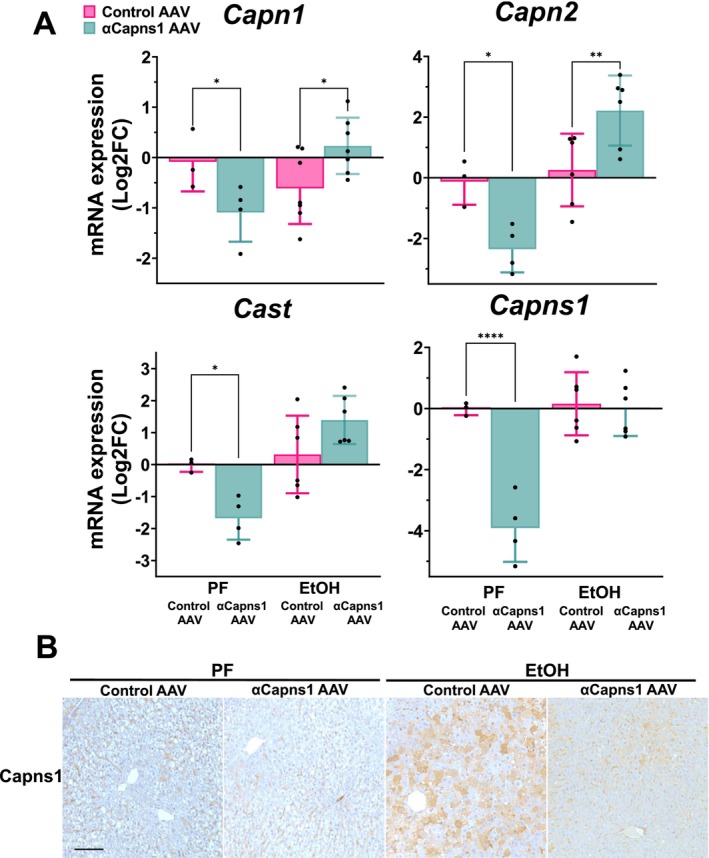
Changes in calpain family expression induced by ethanol exposure: Effect of Capns1 knockdown. Panel (A) Gene expression of Capn1, Capn2, CAST, and Capns1 in wild‐type and Capns1‐knockdown mice. Panel (B) Immunostaining for Capns1. Representative photomicrographs (20×). Scale bar = 100 μm. AAV, adeno‐associated virus; EtOH, ethanol‐fed; PF, pair‐fed. *p < 0.05, **p < 0.01, and ****p < 0.0001.

In pair‐fed group, the expressions of *Capn1*, *Capn2*, *Cast*, and *Capns1* remained unchanged by ethanol exposure at determined by RT‐PCR, suggesting the mRNA expression of these genes are either minimally responsive to ethanol or regulated through alternative pathways. Following *Capns1* knockdown, the expressions of *Capn1* and *Capn2* increased in both pair‐fed and ethanol groups (Figure 2A). In contrast, the response of *Cast* and *Capns1* varied between groups: while its expression remained stable in ethanol group following *Capns1* knockdown, pair‐fed group showed significantly decreased *Cast* and *Capns1* after knockdown (Figure 2A). Parallel to the RT‐PCR results, immunohistochemical detection of Capns1 showed that ethanol exposure enhanced protein levels, which was dramatically attenuated following knockdown (Figure 2B). Taken together, these results indicate that even though transcriptional changes were modest, the αCapns1 AAV effectively prevented both basal Capns1 protein expression and the ethanol‐induced increase, demonstrating successful functional knockdown at the protein level.

### 3.3 Capns1 Knockdown Protected Against Microvesicular Lipid Accumulation Caused by Ethanol Exposure

**FIGURE 3:**
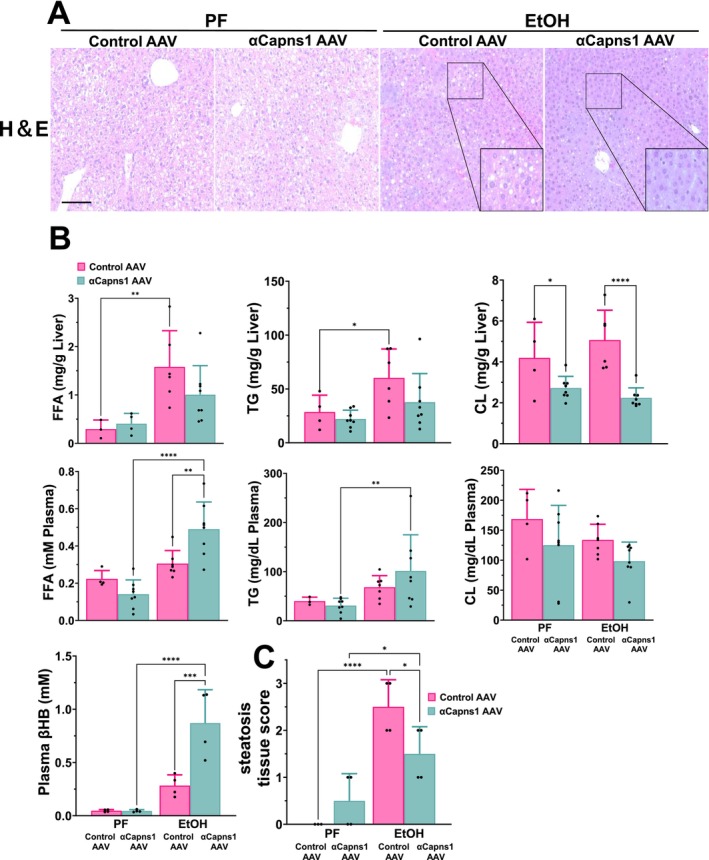
Capns1 knockdown protected against microvesicular lipid accumulation caused by ethanol exposure. Panel (A) Hematoxylin and eosin (H&E) staining of mouse liver sections. Representative photomicrographs (20×). Scale bar = 100 μm. Panel (B) Levels of free fatty acids (FFA), triglycerides (TG), cholesterol (CL), and β‐hydroxybutyrate (βHB) were measured biochemically. Panel (C) Small lipid droplets were scored in H&E‐stained sections to assess microvesicular steatosis. Tissue scores were assigned based on the percentage of microvesicular steatosis observed in a single field as follows: Score 0: 0%; Score 1: 1%–25%; Score 2: 26%–50%; Score 3: 51%–75%; Score 4: > 75%. *p < 0.05, **p < 0.01, ***p < 0.001, and ****p < 0.0001. AAV, adeno‐associated virus; EtOH, ethanol‐fed; PF, pair‐fed.

Ethanol consumption is known to cause hepatic steatosis, characterized by both macro‐ and microvesicular steatosis. Macrovesicular lipid droplets are enriched predominantly in TG (Alamri et al. 2019). In contrast, microvesicular steatosis, characterized by accumulation of small lipid droplets, occurs when mitochondrial β‐oxidation and/or CL metabolism is impaired (Fromenty and Pessayre 1997; Minamikawa et al. 2020). In this study, ethanol exposure significantly increased the accumulation of microvesicular lipid droplets, which was attenuated by Capns1 knockdown (Figure 3A,C). To further characterize the specific effect of ethanol and Capns1 knockdown on lipid accumulation, total levels of TG, CL, and FFA concentrations in the liver and plasma were determined biochemically (Figure 3B). In addition, βHB in the plasma was measured (Figure 3B). Ethanol exposure caused accumulation of all three lipid pools within the liver, but only the accumulation of CL was significantly attenuated by Capns1 knockdown (Figure 3B). In addition, Capns1 knockdown significantly increased FFA and βHB in plasma in the ethanol group (Figure 3B). These results suggest that Capns1 knockdown specifically targets the microvesicular steatosis pathway by reducing hepatic CL accumulation while promoting lipid mobilization and β‐oxidation, accompanied by increased plasma free fatty acids and β‐hydroxybutyrate.

### 3.4 Capns1 Knockdown Enhances the Expression of Cholesterol‐Related Genes

**FIGURE 4:**
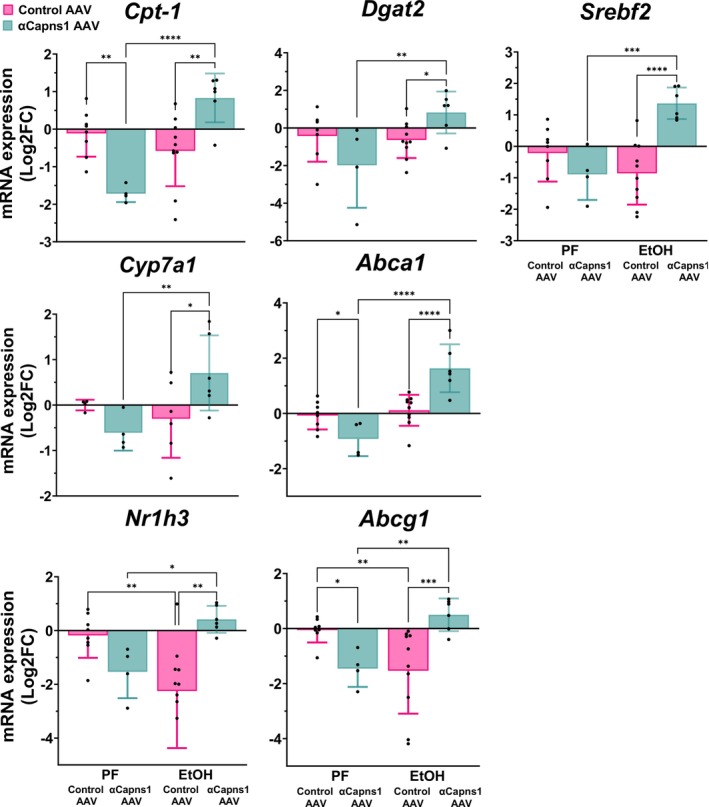
Capns1 knockdown is associated with altered expression of cholesterol‐related genes. The expression of genes involved in fatty acid metabolism (*Cpt1a*), triglyceride synthesis (*Dgat2*), and cholesterol homeostasis (*Cyp7a1*, *Srebf2*, *Nr1h3* encoding LXRα, *Abca1*, and *Abcg1*) was determined using real‐time RT‐PCR. *p < 0.05, **p < 0.01, ***p < 0.001, and ****p < 0.0001. AAV, adeno‐associated virus; PF, pair‐fed; EtOH, ethanol‐fed.

The expression of key lipid metabolism genes was then determined. These included genes involved in fatty acid metabolism (*Cpt1a*, *Fasn*), triglyceride synthesis (*Dgat2*), and CL homeostasis (*Cyp7a1*, *Lcat*, *Srebf2*, *Nr1h3* encoding LXRα, *Abca1*, and *Abcg1*) (Figure 4, See Figure S2A). Ethanol exposure alone did not significantly alter the expression of any of these genes (Figure 4). However, Capns1 knockdown in the ethanol group significantly increased the expression of *Cpt1a* (fatty acid oxidation), *Dgat2* (triglyceride synthesis), *Srebf2* (CL biosynthesis regulation), *Nr1h3* (CL homeostasis), *Abca1* and *Abcg1* (reverse CL transport) compared to the control virus group. This coordinated gene expression pattern indicates that Capns1 knockdown induces coordinated changes in genes that enhance fatty acid oxidation and CL efflux.

### 3.5 Transcriptomic Analysis Reveals Reprogramming of Cholesterol Metabolism by Capns1 Knockdown Under Ethanol Stress

**FIGURE 5:**
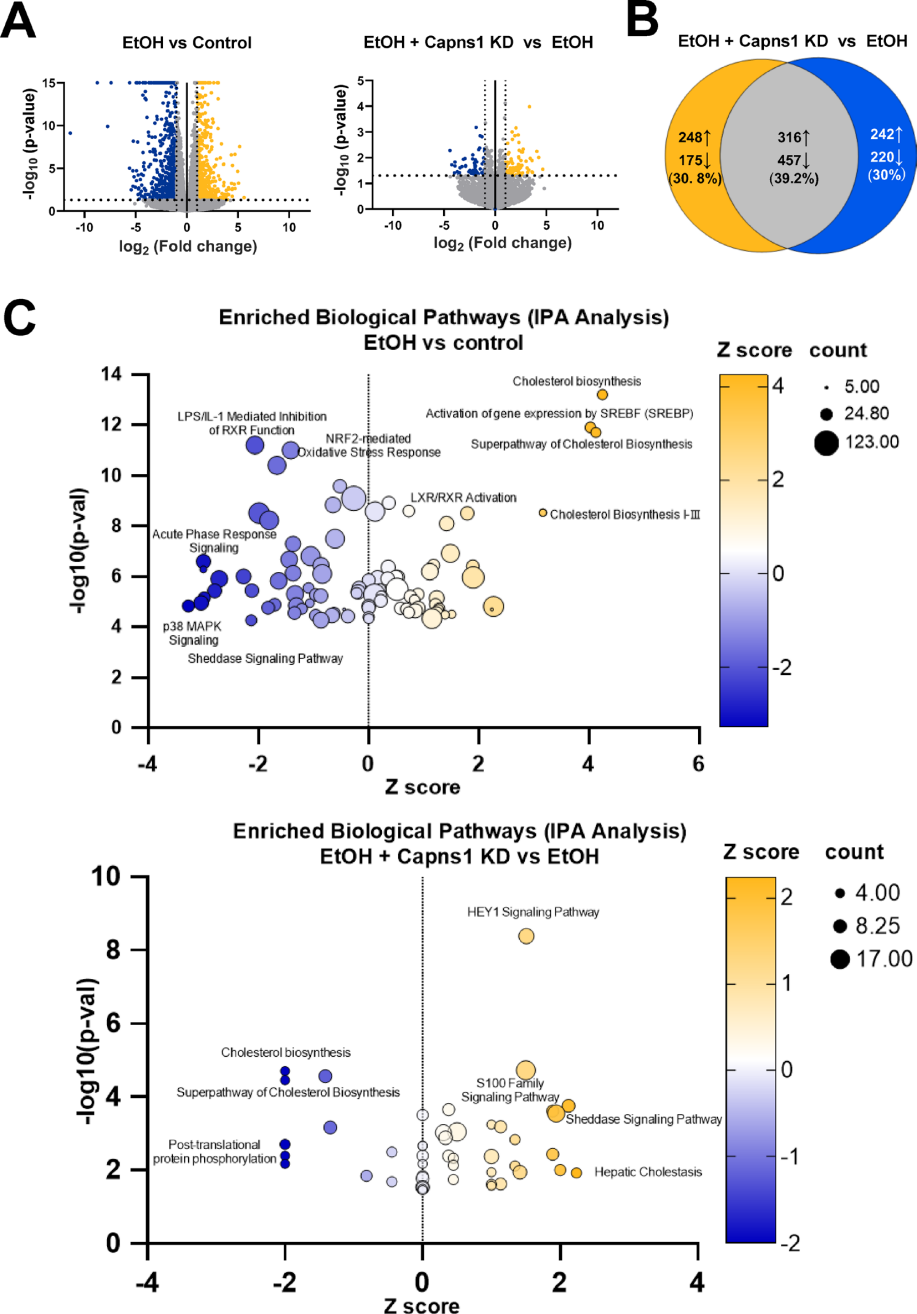
Transcriptomic analysis indicates changes in cholesterol metabolism by Capns1 knockdown under ethanol stress. Panel (A) Volcano plots showing differentially expressed genes (DEGs) in EtOH versus Control (left) and EtOH + Capns1 KD versus EtOH (right). Cutoffs: |log_2_FC| > 1 and *p* < 0.05. Panel (B) Venn diagram illustrating the overlap of DEGs between EtOH + Capns1 knockdown and EtOH. Panel (C) Canonical pathway analysis (IPA) of DEGs in EtOH versus Control and EtOH + Capns1 KD versus EtOH. Among the top 100 canonical pathways ranked by *p*‐value, only those for which an activation *z*‐score could be calculated are presented in the pathway chart. Control, pair‐fed wild‐type mice; IPA, Ingenuity Pathway Analysis; KD, knockdown.

To understand the broader transcriptional consequences underlying these phenotypic and molecular changes, we performed RNA sequencing analysis on hepatic tissue from all treatment groups (Control, EtOH, Capns1 KD, and EtOH+Capns1 KD). Differential expression analysis revealed that ethanol exposure significantly altered hepatic gene expression compared to Control, as shown in the volcano plot (Figure 5A, left panel). Capns1 knockdown under ethanol exposure induced a distinct set of transcriptional changes compared to ethanol treatment alone (Figure 5A, right panel), suggesting that Capns1 modulates the transcriptomic response to ethanol in a calpain‐dependent manner.

Venn diagram analysis demonstrated a modest overlap (39.2% of DEGs shared) between the EtOH+Capns1 KD versus EtOH and EtOH versus Control comparisons, while 30.8% and 30.0% of DEGs were unique to each comparison, respectively (Figure 5B). This pattern indicated that Capns1 knockdown substantially reprograms, rather than simply reverses, ethanol‐induced transcriptional changes. Pathway analysis using IPA revealed clear differences between treatment groups (Figure 5C). While ethanol exposure robustly activated biosynthetic pathways, most notably CL biosynthesis (*Z*‐score > 4), compared to Control, Capns1 knockdown under ethanol exposure suppressed these same CL biosynthetic pathways (*Z*‐score < 0). Additional pathways affected included LXR/RXR activation, which was prominently upregulated by ethanol exposure but showed different regulation patterns following Capns1 knockdown. These results suggest that Capns1‐dependent calpain activity may be involved in the regulation of CL metabolic pathways under ethanol exposure, particularly transcriptional programs that include LXR/RXR signaling. Detailed results of pathway and upstream regulator analyses are provided in Table S4.

### 3.6 Capns1 Knockdown Modulates Cholesterol Metabolic Proteins and Promotes Nuclear Translocation of LXRα

**FIGURE 6:**
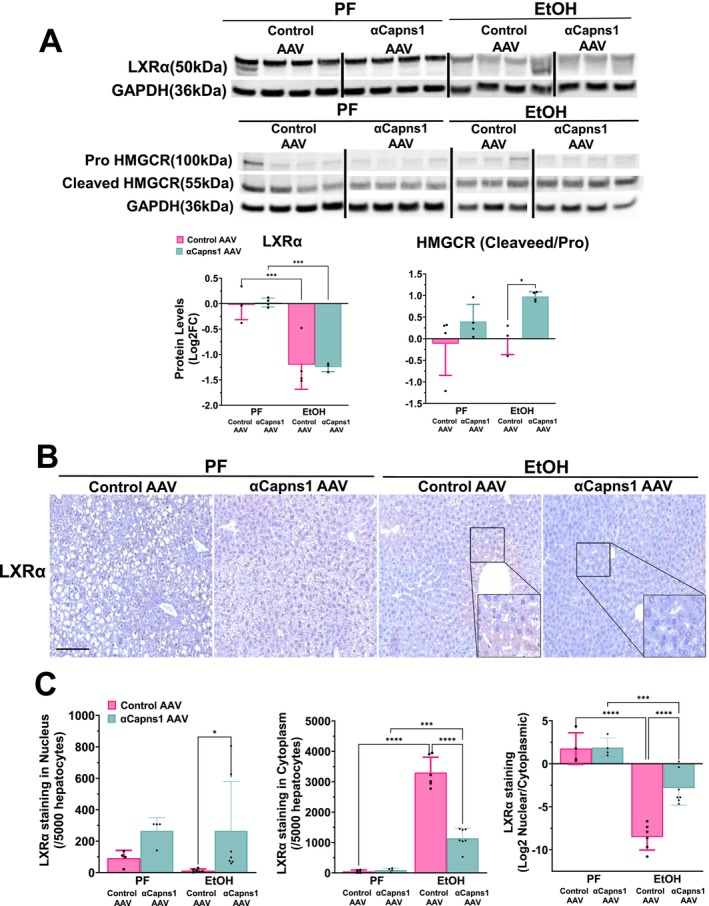
Capns1 knockdown modulates cholesterol metabolic proteins and promotes nuclear translocation of LXRα. Panel (A) Western blot analysis was performed to evaluate the expression of HMGCR and LXRα. Additional data for ABCA1, ABCG1, and VCP are shown (Figure S2B). Panel (B) Immunohistochemical staining of LXRα in liver tissue, which indicates the nuclear and cytoplasmic localization of LXRα, is shown. Representative photomicrographs (20×). Scale bar = 100 μm. Panel (C) We quantified LXRα‐positive nuclei and cytoplasm per 5000 hepatocytes. *p < 0.05, ***p < 0.001, and ****p < 0.0001 AAV, adeno‐associated virus; EtOH, ethanol‐fed; PF, pair‐fed.

To further investigate the mechanism by which Capns1 regulates CL metabolism, we examined protein levels of key mediators involved in this pathway, specifically focusing on LXRα and HMGCR expression (Figure 6). Ethanol exposure significantly altered LXRα protein expression; however, this ethanol‐induced change was not affected by Capns1 knockdown. We also analyzed HMGCR, the rate‐limiting enzyme in CL biosynthesis, which exists in two distinct forms with different enzymatic activities. The enzymatically active form (Pro‐HMGCR) can be processed by proteases to generate cleaved forms, which may exhibit altered enzymatic activity and thereby serve as a regulatory mechanism for HMGCR function (Burg and Espenshade 2011; Omkumar et al. 1992). Notably, Capns1 knockdown mice showed a significantly elevated ratio of cleaved/pro‐HMGCR, which is consistent with reduced functional HMGCR activity and indicative of reduced CL biosynthesis. Additional protein expressions were also measured and are presented in Figure S2B.

Although ethanol exposure significantly decreased LXRα protein expression, no significant change was observed at the mRNA level (Figures 4 and 6A). To assess the functional implications of these protein‐level changes, we examined LXRα subcellular localization using immunohistochemical staining. LXRα is a nuclear receptor that requires nuclear localization for transcriptional activity (Prufer and Boudreaux 2007). In this study, ethanol exposure increased cytoplasmic localization of LXRα in hepatocytes (Figure 6B,C). In contrast, following Capns1 knockdown, the localization of LXRα changed, with decreased cytoplasmic localization and increased nuclear localization (Figure 6B,C).

## 4 Discussion

The present study reveals that Capns1 knockdown exerts notable effects on hepatic lipid metabolism in experimental ALD. Although ALT and *Pai1* expression did not differ significantly between groups (Figure 1), suggesting that Capns1 knockdown does not strongly affect acute inflammatory signaling in this model, the present study revealed intriguing metabolic effects of calpain inhibition. Modest changes in macrophage‐associated markers were observed; however, these were not accompanied by alterations in injury markers, supporting a limited inflammatory impact. In particular, the impact of Capns1 knockdown on hepatic lipid metabolism—including CL homeostasis and β‐oxidation—emerged as a central finding. Detailed evaluation of inflammatory responses is provided in the Supporting Information Discussion.

**FIGURE 7:**
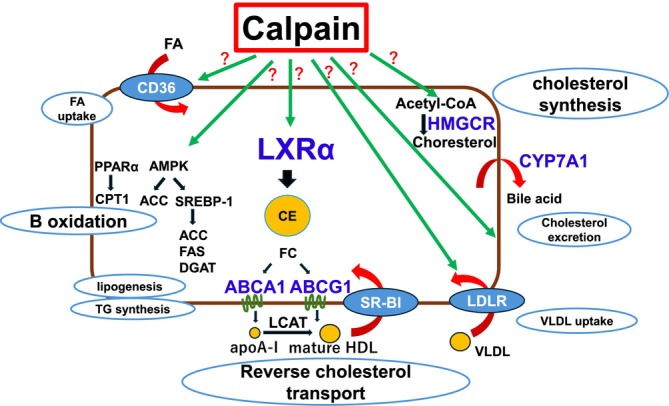
Proposed model linking Capns1 to cholesterol metabolism in ethanol‐treated liver. Schematic illustration summarizing the proposed role of Capns1‐dependent calpain activity in regulating hepatic cholesterol metabolism during ethanol exposure. Ethanol feeding is associated with dysregulated calpain signaling, altered LXRα subcellular localization, and activation of cholesterol biosynthetic pathways. Capns1 knockdown suppresses ethanol‐induced cholesterol biosynthesis, promotes LXRα nuclear translocation, and increases the cleaved/pro‐HMGCR ratio, thereby limiting functional HMGCR availability. Green arrows indicate regulatory relationships between Calpain and downstream targets. Question marks (?) denote connections that are proposed based on the collective data but for which the direct molecular mechanisms remain to be established. Collectively, these changes contribute to improved cholesterol homeostasis and are associated with reduced hepatic cholesterol accumulation in ethanol‐treated mice.

Given these findings, we focused our analysis on lipid metabolism, particularly CL homeostasis. Hepatic lipid metabolism is regulated through multiple interconnected pathways, including fatty acid uptake, synthesis, oxidation, and transport. Within this broad metabolic network, CL metabolism is subject to particularly complex and multilayered regulatory control, maintained through tightly coordinated regulation of three major processes: synthesis, uptake, and efflux. Prior to the current work, the specific molecular steps within this network targeted by calpain had not been defined; our findings point to HMGCR and LXRα as key nodes of calpain‐mediated regulation (Figure 7). Our data demonstrate that Capns1 knockdown selectively attenuates CL accumulation with no significant change in hepatic TG or FFA levels (Figure 3). This selectivity is notable because, while ethanol is known to promote hepatic lipogenesis and concurrently suppress β‐oxidation, the impact of ethanol on CL metabolism remains inconclusive (Hoebinger et al. 2023; You and Arteel 2019). The selective effect on CL suggests specific involvement of calpain in CL metabolism within the context of ALD, with HMGCR and LXRα emerging as key nodes of regulation (Figure 7). Additionally, Capns1 knockdown markedly attenuated ethanol‐induced microvesicular steatosis (Figure 3), indicating that changes in CL metabolism may contribute to the amelioration of lipid droplet accumulation in the liver. This interpretation is consistent with the established role of CL accumulation in the pathogenesis of microvesicular steatosis. Taken together, the selective reduction in hepatic CL by Capns1 knockdown is likely to be a primary driver of the observed improvement in microvesicular steatosis.

To gain mechanistic insight into these phenotypic changes, RNA‐seq analysis was performed, which revealed that Capns1 knockdown suppressed the ethanol‐induced activation of CL biosynthetic pathways in ethanol‐treated mice. Previous studies have demonstrated the indispensable regulatory role of Capns1 (Arthur et al. 2000). Consistent with this, Capns1 knockdown alone exerted marked effects on multiple metabolic regulatory pathways in the present study (see Table S4). Under ethanol exposure, however, Capns1 knockdown reprogrammed hepatic gene expression, supporting its critical role in maintaining CL homeostasis during alcohol‐induced liver injury. Because the canonical pathway analysis used in IPA evaluates coordinated transcriptional changes across an entire biological pathway rather than focusing on individual gene alterations, *Hmgcr* and *Nr1h3* did not emerge as significant DEGs in our RNA‐seq dataset. Nevertheless, CL‐related pathways—including CL biosynthesis, activation of gene expression by *Srebf*, and LXR/RXR Activation—were among the top enriched pathways, and *Srebf2* was also identified as a predicted upstream regulator (see Table S4). These results indicate that CL homeostasis is regulated at the pathway level, even when changes in individual genes do not reach statistical significance, and support our experimental findings.

We observed increased expression levels of genes involved in CL homeostasis, including *Srebf2*, *Cyp7a1*, *Nr1h3* (encoding LXRα), *Abca1*, and *Abcg1*, in Capns1‐knockdown mice (Figure 4). This upregulation may reflect a compensatory response to decreased intracellular CL levels, consistent with sterol‐sensitive regulation of SREBP signaling and LXR‐dependent CL homeostatic programs (Goldstein et al. 2006; Schulman 2017). The reduction in hepatic CL levels reported in Figure 3B was measured in homogenates of rinsed livers, from which residual blood and extracellular fluid had been removed; this measurement therefore reflects the intracellular CL pool. These data are thus consistent with the compensatory transcriptional response observed in Figure 4. Future studies employing subcellular fractionation to resolve compartment‐specific CL distribution will be needed to further characterize this mechanism. SREBP2 promotes CL biosynthesis via HMGCR and uptake via LDLR (Madison 2016; Marquart et al. 2010). Interestingly, despite increased Srebf2 expression in Capns1‐knockdown mice, the increased cleaved/pro‐HMGCR ratio suggests reduced functional HMGCR availability, consistent with decreased biosynthetic capacity. Previous studies have demonstrated that HMGCR can be proteolytically cleaved by Capn2, suggesting that HMGCR is a substrate of calpains (Parker et al. 1984). Much of HMGCR degradation is thought to be regulated by the ubiquitin–proteasome system, which operates in response to intracellular CL levels (Shi et al. 2022). To clarify the involvement of calpains, further investigation of the broader regulatory network—including the HMGCR degradation pathway—is warranted.

In addition to effects on CL biosynthesis, Capns1 knockdown appears to influence CL efflux. *Nr1h3* encodes LXRα, which facilitates reverse CL transport (RCT) by promoting CL efflux via Abca1 and Abcg1, as well as by inducing bile acid synthesis via *Cyp7a1* (Naik et al. 2006; Peet et al. 1998; Wagner et al. 2003). Our findings suggest that Capns1 may influence the RCT pathway via LXRα. Supporting this interpretation, LXRα nuclear translocation was enhanced in Capns1‐knockdown livers, as shown by immunostaining. Because LXRα must localize to the nucleus to activate transcription of its target genes (Miller et al. 2009; Prufer and Boudreaux 2007), its nuclear translocation is considered important for RCT induction. The nuclear import of LXRα has been reported to involve a basic amino acid‐rich nuclear localization signal and a heterodimeric partner nuclear receptor, and previous studies have reported that this partner may also serve as a substrate of Capn2 (Matsushima‐Nishiwaki et al. 1996; Prufer and Boudreaux 2007). In addition, LXRα itself has been proposed as a calpain substrate in macrophages (Miyazaki and Miyazaki 2017). However, the precise molecular mechanisms underlying LXRα nuclear translocation and the potential direct involvement of calpain in this process remain to be elucidated.

Beyond its effects on CL metabolism, Capns1 knockdown was associated with increased expression of *Cpt1a*, the rate‐limiting enzyme in mitochondrial β‐oxidation, which may counteract ethanol‐mediated suppression of fatty acid oxidation (Figure 4). Mitochondrial dysfunction is a key contributor to impaired β‐oxidation in liver disease (Wajner and Amaral 2015). The elevated serum FFA levels observed in Capns1‐knockdown mice may reflect enhanced hepatic lipid export, potentially as a consequence of improved β‐oxidation. In addition, the concurrent upregulation of *Dgat2* may represent a compensatory response to transient lipotoxic stress during transient FFA elevation (Listenberger et al. 2003). Moreover, ketone bodies are primarily produced in the liver from acetyl‐CoA derived from β‐oxidation and are subsequently transported to extrahepatic tissues for final oxidation (Puchalska and Crawford 2017). In nonalcoholic fatty liver disease, mitochondrial dysfunction is known to impair β‐oxidation, leading to decreased serum β‐hydroxybutyrate (βHB) levels (Moore et al. 2024). In this study, increased βHB levels in Capns1‐knockdown mice are associated with improved mitochondrial β‐oxidation. Therefore, Capns1 knockdown may contribute to improved mitochondrial function.

These mitochondrial changes may influence lipid droplet composition. Early‐stage ALD is characterized by the accumulation of LDs containing TG and cholesteryl esters (CE) (Gao and Bataller 2011; Mashek et al. 2015). Notably, there are two types of LDs: LDs‐TG‐rich and CE‐rich, each with distinct protein profiles (Khor et al. 2014). Previous studies have demonstrated that LDs observed in microvesicular steatosis are predominantly enriched in CL rather than TG (Minamikawa et al. 2020). Moreover, hepatic CL overload has been shown to induce microvesicular steatosis and concurrently impair mitochondrial function, suggesting a link between CL accumulation and mitochondrial dysfunction in the liver (Dominguez‐Perez et al. 2019).

The increased expression of lipid biosynthesis‐related genes observed in the ethanol group with Capns1 knockdown (Figure 4) does not necessarily indicate enhanced TG synthesis. Fasn gene expression did not show a significant change in Capns1‐knockdown mice (Figure S2), and RNA‐seq analysis also did not indicate strong involvement of TG synthesis‐related pathways (Table S4). These findings suggest that Capns1 is unlikely to directly regulate TG synthesis.

In ALD, mitochondrial impairment contributes to excessive reactive oxygen species generation and endoplasmic reticulum stress (Garcia‐Ruiz et al. 2013) processes that may be further exacerbated by ethanol‐induced disruption of CL metabolism. Our findings suggest that Capns1 knockdown alters hepatic metabolic responses to ethanol exposure, particularly affecting lipid metabolism centered on LXRα and HMGCR (Figure 7), accompanied by mitochondrial‐related changes and reduced microvesicular steatosis in ethanol‐treated mice. Hepatic lipid metabolism involves multiple intersecting pathways, and the pathogenesis of ALD is highly complex. Numerous calpain substrates remain unidentified. It should be noted that the cleaved/Pro‐HMGCR ratio assessed by western blotting represents an indirect measure of HMGCR activity, and that HMGCR is subject to regulation through multiple posttranslational mechanisms; therefore, changes in protein processing alone are insufficient to fully characterize the functional state of HMGCR. Furthermore, whether the change in LXRα nuclear localization observed in this study reflects a direct effect of calpain‐mediated proteolysis on the nuclear translocation machinery, or rather represents a secondary consequence of altered CL metabolism driven by Capns1 knockdown, remains to be determined in future studies. Given that Capns1 serves as the regulatory subunit of calpain and modulates its proteolytic activity toward specific substrates, Capns1 may influence LXRα nuclear translocation and HMGCR stability through calpain‐mediated limited proteolysis of their regulatory factors. Further investigation into whether RXRα and other LXRα‐associated molecules serve as potential calpain substrates will be warranted to elucidate these calpain‐regulated molecular mechanisms, which may ultimately contribute to clarifying ALD pathogenesis and identifying novel therapeutic targets for restoring CL homeostasis and preventing disease progression.

## Funding

This work was supported by the National Institutes of Health, R01DK130294, R01AA028436, and P30DK120531.

## Conflicts of Interest

The authors declare no conflicts of interest.

## Supporting information


**Figure S1:** Effect of ethanol exposure and Capns1 knockdown on indices of liver injury.

**Figure S2:** Effect of ethanol exposure and Capns1 knockdown on lipid metabolism.

**Figure S3:** Canonical pathways and upstream regulators identified by Ingenuity Pathway Analysis (IPA) in each group comparison from the RNA‐seq dataset.

**Table S1:** Product information for primers used in RT‐PCR.
**Table S2:** Drugs and chemical assays used in this study.
**Table S3:** Primary antibodies used in this study.
**Table S4:** Enriched canonical pathways and upstream regulators (IPA analysis).

## Data Availability

The human RNA‐seq datasets analyzed in this study are available in the Database of Genotypes and Phenotypes (dbGAP, accession: phs001807.v1.p1). All other data supporting the findings of this study are available from the corresponding author upon reasonable request.
