# Supplementary figures and images for: Calpain‐4 Knockdown Modulates Cholesterol Metabolism and LXRα Nuclear Localization in Experimental Alcohol‐Related Liver Disease
Source: Alcohol Clin Exp Res (Hoboken). 2026 Jun 17;50(6):e70356. doi: 10.1111/acer.70356 (PMC13273926; doi:10.1111/acer.70356)

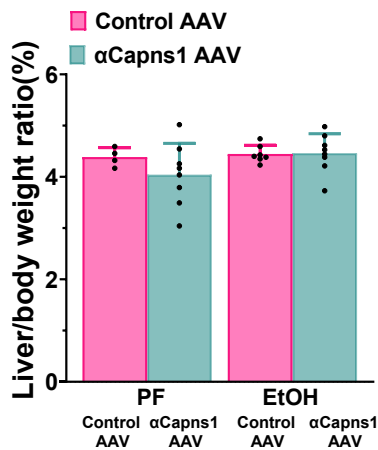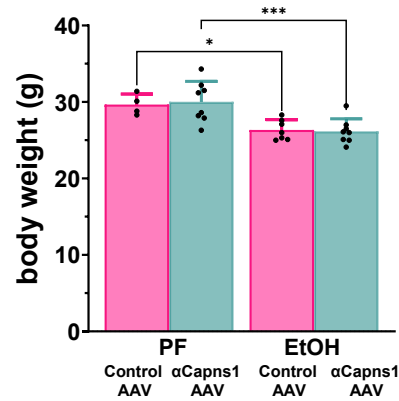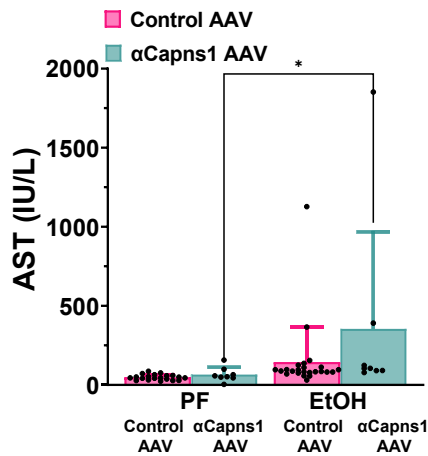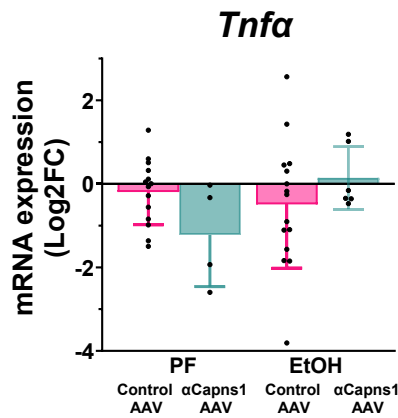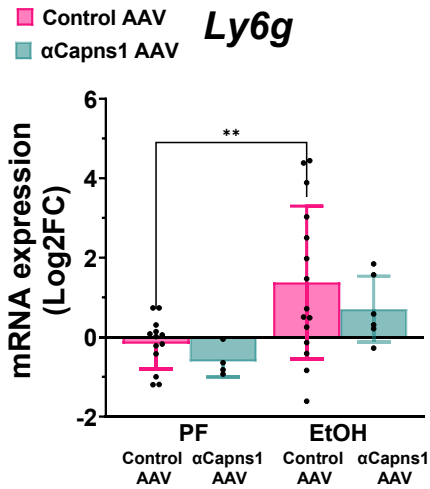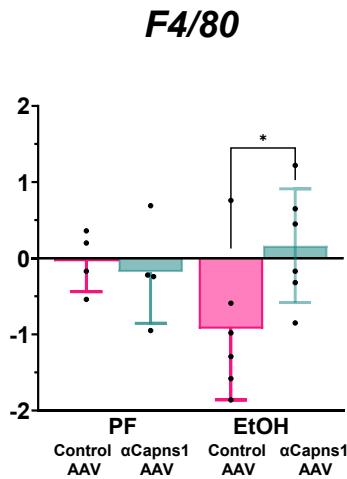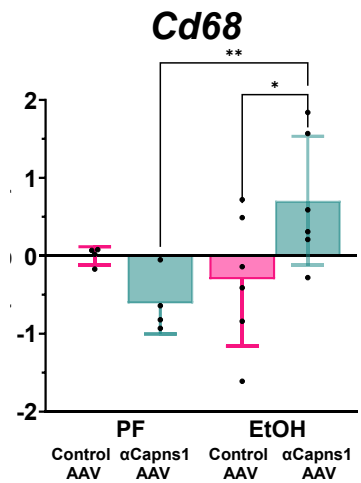

Supplement: Supplementary file 1 — Figure S1: Effect of ethanol exposure and Capns1 knockdown on indices of liver injury. [file ACER-50-0-s001.pdf]

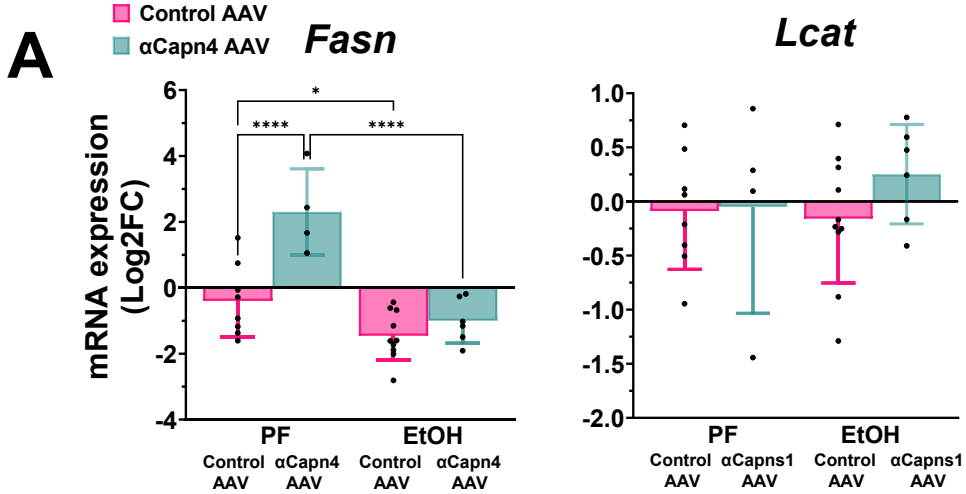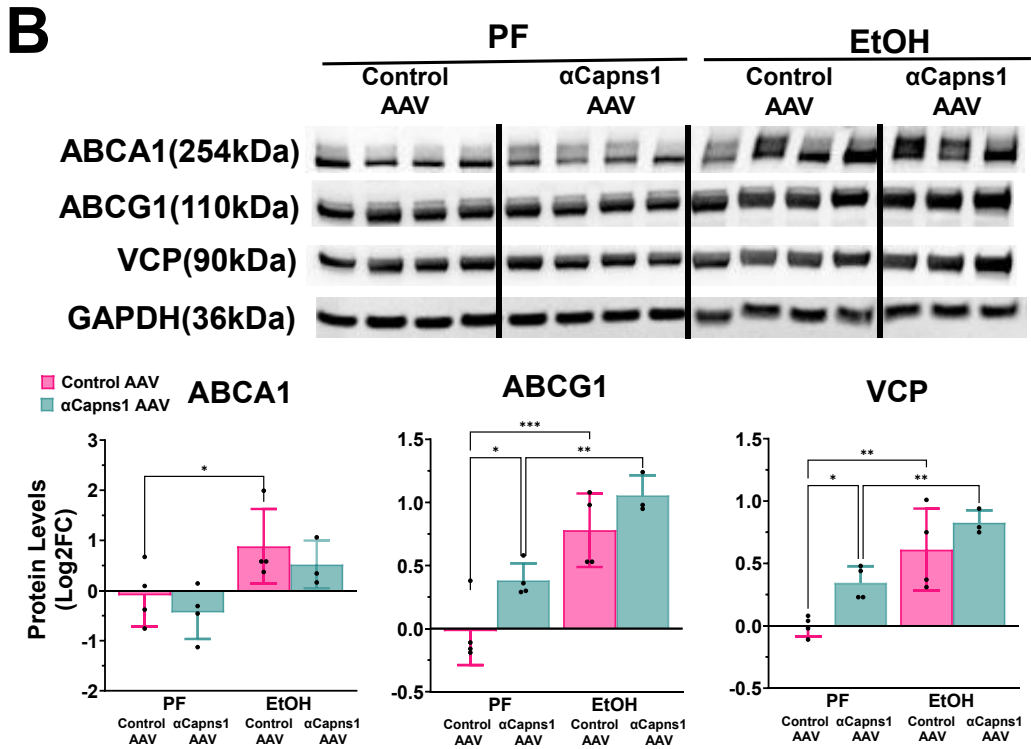

Supplement: Supplementary file 2 — Figure S2: Effect of ethanol exposure and Capns1 knockdown on lipid metabolism. [file ACER-50-0-s002.pdf]

# Enriched Biological Pathways (IPA Analysis) Capns1 KD vs control

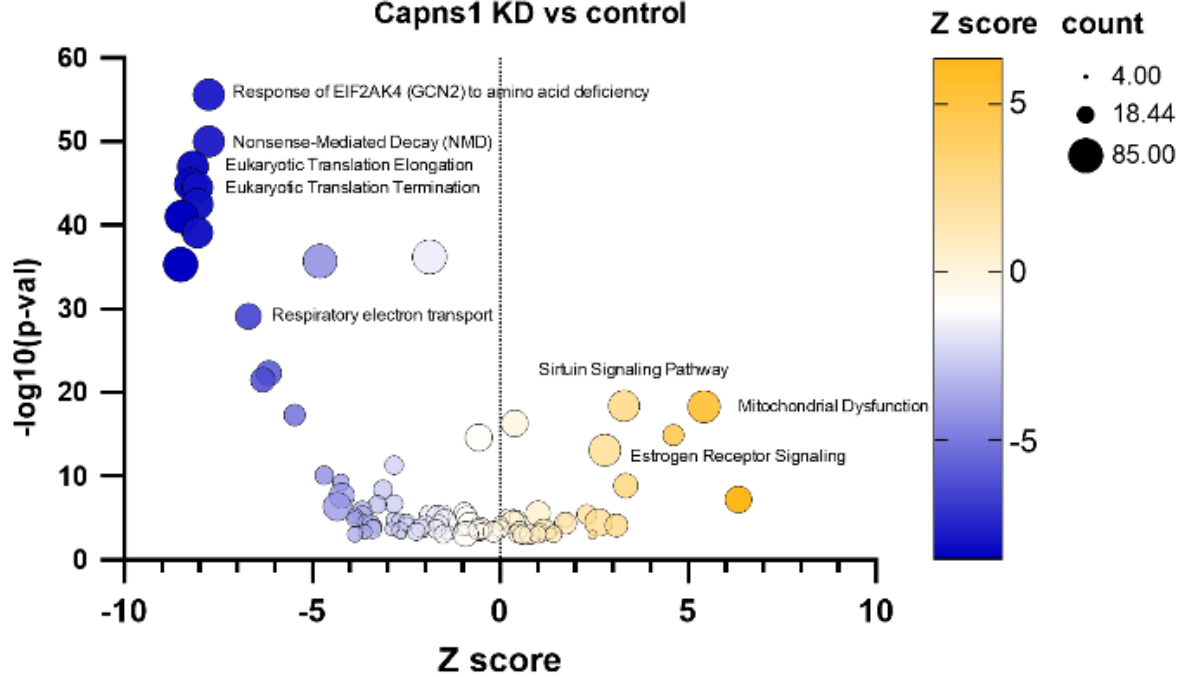

Supplement: Supplementary file 3 — Figure S3: Canonical pathways and upstream regulators identified by Ingenuity Pathway Analysis (IPA) in each group comparison from the RNA‐seq dataset. [file ACER-50-0-s004.pdf]
