# Supplementary material for: Calpain‐4 Knockdown Modulates Cholesterol Metabolism and LXRα Nuclear Localization in Experimental Alcohol‐Related Liver Disease
Source: Alcohol Clin Exp Res (Hoboken). 2026 Jun 17;50(6):e70356. doi: 10.1111/acer.70356 (PMC13273926; doi:10.1111/acer.70356)
Supplement: Supplementary file 4 — Table S1: Product information for primers used in RT‐PCR. Table S2: Drugs and chemical assays used in this study. Table S3: Primary antibodies used in this study. Table S4: Enriched canonical pathways and upstream regulators (IPA analysis). [file ACER-50-0-s003.docx]

SUPPLEMENTARY MATERIAL

Calpain-4 knockdown modulates cholesterol metabolism and LXRα nuclear localization in experimental alcohol-related liver disease

Noriko Kitano^1^, Jiang Li,^1^ Sam Taborski^1^, Charis-Marie Vanderpuye^1^, Pooja Muddasani^1^, Sudrishti Chaudhary^1^, Jia-Jun Liu^4,5^, Silvia Liu^2,4,5^, Juliane I Beier^1,2,3^, Josepmaria Argemi^1,6^, Ramon Bataller^7^, and Gavin E Arteel^1,2^

^1^Department of Medicine, Division of Gastroenterology, Hepatology and Nutrition, ^2^Pittsburgh Liver Research Center, University of Pittsburgh, ^3^Department of Environmental and Occupational Health, ^4^Pharmacology and Chemical Biology, University of Pittsburgh, ^5^Organ Pathobiology and therapeutics institute, University of Pittsburgh, ^6^ Department of Internal Medicine, Liver Unit, Clinical University of Navarra, Navarra, Spain, ^7^Institut d’Investigacions Biomediques August Pi i Sunyer (IDIBAPS), University of Barcelona, Barcelona, Spain.

Send all correspondence to: Gavin E. Arteel, PhD, FAASLD

Thomas E. Starzl Biomedical Science Tower

West 1144

200 Lothrop Street

Pittsburgh, PA 15213

Phone: +1-412-648-4187

Email: gearteel@pitt.edu

**Supplementary Experimental Procedures**

Information on RT-PCR primers and probes, chemical assays, and antibodies used in this study are summarized in Supplementary Tables 1–3.
In addition, Supplementary Table 4 lists the top canonical pathways and upstream regulators identified by Ingenuity Pathway Analysis (IPA) for each of the three group comparisons (EtOH vs Control, Capns1KD vs Control, and EtOH+Capns1KD vs EtOH) from the RNA-seq dataset. Pathways are ranked by –log10 p-value, and z-scores are provided where available to indicate predicted activation or inhibition.

**Supplementary Results**

Effect of ethanol exposure and Capns1 knockdown on indices of liver injury.

In addition to body weight and liver weight, we measured serum AST levels, the expression of proinflammatory gene *(Tnfα*), and immune cell markers including*Ly6g* (neutrophils), *F4/80* (Kupffer cells and monocytes), and *Cd68*(inflammatory macrophages) to assess liver injury. Ethanol exposure significantly reduced body weight gain, whereas no significant differences in the liver weight–to–body weight ratio were observed between groups. Although ethanol exposure has been reported to increase serum AST levels and inflammatory gene expression, these changes did not reach statistical significance in the present study (Figure S1).

Kupffer cells are known to respond to inflammatory stimuli by releasing hepatotoxic mediators such as reactive oxygen species and cytokines, which can contribute to neutrophil recruitment.

As a result, the activation peaks of neutrophils and Kupffer cells do not necessarily coincide. These factors may have complicated the evaluation of ethanol-induced inflammatory responses.

In our experimental results, Capns1 knockdown did not markedly affect inflammatory markers in ALD; however, our metabolic analyses suggested improvements in hepatic lipid metabolism along with enhanced β-oxidation and mitochondrial function. We observed modest increases in Cd68 and F4/80 expression in the Capns1 knockdown group; however, these changes were not accompanied by alterations in injury markers or inflammatory cytokines, suggesting limited biological impact.

Macrophage-associated pathways have been implicated in lipid metabolic processes, including β-oxidation. Taken together, these observations may indicate subtle alterations in macrophage-associated gene expression; however, additional studies are required to determine whether these changes reflect shifts in macrophage phenotype associated with altered metabolic regulation. Further studies are warranted to clarify how calpain modulates macrophage-dependent lipid metabolism in the context of ALD.

Effect of ethanol exposure and Capns1 knockdown on lipid metabolism.

We examined genes involved in lipid metabolism, including*Fasn* and *Lcat*. Despite prior findings that ethanol exposure promotes lipogenesis via upregulation of *Fasn*, there were no significant differences in this study. Capns1 knockdown in ethanol group did not alter the expression of these genes (Figure S2A). In addition, we analyzed proteins related to cholesterol metabolism, including VCP, ABCA1 and ABCG1. VCP is critical for the ER-associated degradation of HMG-CoA reductase and has been reported as a calpain substrate. In macrophages, it has been also reported that ABCA1 and ABCG1 are substrates of calpain. However, despite prior reports linking calpain activity to the regulation of these proteins, Capns1 knockdown in ethanol group did not alter their expression, suggesting that calpain-dependent regulation of these proteins may be context- or cell type–specific.

Transcriptomic analysis reveals reprogramming of cholesterol metabolism by Capns1 knockdown under ethanol stress.

To complement the primary findings presented in the main manuscript, we provide supplementary canonical pathway and upstream regulator analyses for the three group comparisons: EtOH vs Control, Capns1KD vs Control, and EtOH+Capns1KD vs EtOH.

These analyses revealed broader transcriptomic alterations in addition to the cholesterol metabolism pathways emphasized in the main text. In the EtOH vs Control comparison, oxidative stress-related pathways—including NRF2-mediated Oxidative Stress Response (z-score: –1.41) and Acute Phase Response Signaling (z-score: –3.00)—were suppressed, in addition to the marked activation of biosynthetic programs such as Cholesterol Biosynthesis (z-score: 4.24). These transcriptomic changes are consistent with an oxidative stress–associated response to ethanol exposure.

In the Capns1KD vs Control comparison, there was profound suppression of anabolic programs related to glucose and lipid metabolism (MLXIPL, z-score: –7.84), as well as protein synthesis pathways such as Response of EIF2AK4 (GCN2) to amino acid deficiency (z-score: –7.76) and Eukaryotic Translation Initiation (z-score: –8.25) (Supplementary table 4). Capns1 encodes the essential small subunit shared by classical calpains (calpain-1 and calpain-2), and its genetic deletion results in embryonic lethality before E11.5 in mice, underscoring its indispensable regulatory role. Consistent with this, Capns1 knockdown alone profoundly affected anabolic responses and broad metabolic regulation in this study, supporting its role as a critical regulator (Supplementary Table 4).

In contrast, the EtOH+Capns1KD vs EtOH comparison showed no significant changes in the anabolic responses observed in Capns1KD vs Control, but instead demonstrated suppression of Cholesterol biosynthetic pathways and upstream regulators previously activated by ethanol exposure. These findings provide evidence that cholesterol metabolism undergoes reprogramming in response to ethanol exposure.

**Supplementary Table 1-Product information for primers used in RT-PCR**

| **Gene name** | **Supplier** | **Cat. No.** |
| --- | --- | --- |
| *Capn1* | Thermofisher | Mm00482964_m1 |
| *Capn2* | Thermofisher | Mm00486669_m1 |
| *CapnS1* | Thermofisher | Mm00501568_m1 |
| *Cast* | Thermofisher | Mm01345276_Mh |
| *Tnfa* | Thermofisher | Mm00443258_m1 |
| *Serpine* | Thermofisher | Rn01481341_m1 |
| *Ly6g* | Thermofisher | Mm04934123_m1 |
| *Adgre1* | Thermofisher | Mn00802529_m1 |
| *Cd68* | Thermofisher | Mm03047343_m1 |
| *Srebf2* | Thermofisher | Mm01306292_m1 |
| *Fasn* | Thermofisher | Mm00662319_m1 |
| *Cpt1a* | Thermofisher | Mm00550438_m1 |
| *Dgat2* | Thermofisher | Mm00499536_m1 |
| *Lcat* | Thermofisher | Mm01178820_m1 |
| *Cyp7a1* | Thermofisher | Mm00484150_m1 |
| *Nr1h3* | Thermofisher | Mm00443451_m1 |
| *Abca1* | Thermofisher | Mm01178820_m1 |
| *Abcg1* | Thermofisher | Mm00437390_m1 |

**Supplementary Table 2-** **Drugs and Chemical assays used in this study**

| **Category** | **Name** | **Supplier** | **Cat No.** |
| --- | --- | --- | --- |
| Chemical assays | AST assay kit | Thermofisher | TR70121 |
|  | ALT assay kit | Thermofisher | TR71121 |
|  | Infinity™ Triglycerides Reagent | Thermofisher | TR22421 |
|  | Infinity™ Cholesterol Reagent | Thermofisher | TR13421 |
|  | Free Fatty Acid Assay Kit | Cell Biolabs, Inc. | STA-618 |
|  | Beta-Hydroxybutyrate Assay Kit (Colorimetric) | Abcam | ab83390 |

**Supplementary Table 3-** **Primary antibodies used in this study**

| **Antibody** | **Supplier** | **Cat No.** |
| --- | --- | --- |
| GAPDH | CELL SIGNALING | 5174S |
| Capns1 | LSBio | LS-C482635-30 |
| LXRα | Abcam | ab176323 |
| ABCA1 | Abcam | ab18180 |
| ABCG1 | Proteintech | 13578-1-AP |
| HMGCR | Invitrogen | XL3767433A |
| VCP | Proteintech | 10736-1-AP |

**Supplementary Table 4-** **canonical pathways and upstream regulators (IPA analysis).**

**EtOH vs control -** **canonical pathways**

| Ingenuity Canonical Pathways | -log(p-value) | z-score | gene count |
| --- | --- | --- | --- |
| Cholesterol biosynthesis | 13.2 | 4.243 | 18 |
| Activation of gene expression by SREBF (SREBP) | 11.9 | 4.025 | 21 |
| Superpathway of Cholesterol Biosynthesis | 11.7 | 4.123 | 18 |
| LPS/IL-1 Mediated Inhibition of RXR Function | 11.2 | -2.065 | 62 |
| NRF2-mediated Oxidative Stress Response | 11 | -1.414 | 58 |
| Pulmonary Fibrosis Idiopathic Signaling Pathway | 10.4 | -1.664 | 64 |
| Regulation of lipid metabolism by PPARalpha | 9.57 | -0.522 | 33 |
| Molecular Mechanisms of Cancer | 9.09 | -0.271 | 123 |
| Phase I - Functionalization of compounds | 8.91 | 0.365 | 31 |
| Thrombin Signaling | 8.83 | -0.649 | 47 |
| Role of BRCA1 in DNA Damage Response | 8.59 | 0.728 | 25 |
| RHO GTPase cycle | 8.57 | 0.115 | 75 |
| Cholesterol Biosynthesis I | 8.52 | 3.162 | 10 |
| Cholesterol Biosynthesis II (via 24,25-dihydrolanosterol) | 8.52 | 3.162 | 10 |
| Cholesterol Biosynthesis III (via Desmosterol) | 8.52 | 3.162 | 10 |
| Cardiac Hypertrophy Signaling (Enhanced) | 8.51 | -1.99 | 85 |
| LXR/RXR Activation | 8.5 | 1.789 | 33 |
| Hepatic Fibrosis Signaling Pathway | 8.22 | -1.807 | 70 |
| Aryl Hydrocarbon Receptor Signaling | 8.09 | 1.414 | 40 |
| RAR Activation | 7.49 | -0.611 | 70 |
| Glycation Signaling Pathway | 7.29 | -1.372 | 44 |
| Protein Kinase A Signaling | 6.91 | 1.483 | 64 |
| Neutrophil degranulation | 6.8 | -1.053 | 73 |
| Role of Osteoclasts in Rheumatoid Arthritis Signaling Pathway | 6.67 | -1.443 | 54 |
| Acute Phase Response Signaling | 6.59 | -3 | 38 |
| Extracellular matrix organization | 6.44 | 1.177 | 26 |
| Chronic Myeloid Leukemia Signaling | 6.43 | -0.866 | 49 |
| GP6 Signaling Pathway | 6.4 | 1.89 | 29 |
| FXR/RXR Activation | 6.36 | 0.354 | 38 |
| Response of EIF2AK1 (HRI) to heme deficiency | 6.28 | -3 | 9 |

**Capns1 KD vs control -** **canonical pathways**

| Ingenuity Canonical Pathways | -log(p-value) | z-score | gene count |
| --- | --- | --- | --- |
| Response of EIF2AK4 (GCN2) to amino acid deficiency | 55.6 | -7.761 | 69 |
| Nonsense-Mediated Decay (NMD) | 50 | -7.761 | 69 |
| Eukaryotic Translation Elongation | 47 | -8.185 | 68 |
| Eukaryotic Translation Initiation | 44.9 | -8.25 | 73 |
| Eukaryotic Translation Termination | 44.5 | -8.062 | 66 |
| Selenoamino acid metabolism | 42.5 | -8.066 | 70 |
| Major pathway of rRNA processing in the nucleolus and cytosol | 41 | -8.488 | 77 |
| SRP-dependent cotranslational protein targeting to membrane | 39.1 | -8.062 | 66 |
| Signaling by ROBO receptors | 36.2 | -1.886 | 83 |
| EIF2 Signaling | 35.7 | -4.802 | 81 |
| Ribosomal Quality Control Signaling Pathway | 35.3 | -8.51 | 85 |
| Respiratory electron transport | 29.1 | -6.708 | 45 |
| Oxidative Phosphorylation | 22.3 | -6.164 | 43 |
| Mitochondrial translation | 21.5 | -6.325 | 40 |
| Sirtuin Signaling Pathway | 18.4 | 3.286 | 67 |
| Mitochondrial Dysfunction | 18.3 | 5.416 | 73 |
| Complex I biogenesis | 17.3 | -5.477 | 30 |
| Regulation of eIF4 and p70S6K Signaling | 16.3 | 0.378 | 49 |
| Granzyme A Signaling | 14.9 | 4.6 | 29 |
| mTOR Signaling | 14.6 | -0.577 | 50 |
| Estrogen Receptor Signaling | 13.1 | 2.777 | 71 |
| Cytoprotection by HMOX1 | 11.3 | -2.828 | 22 |
| Mitochondrial protein import | 10.1 | -4.69 | 22 |
| Complex IV assembly | 9.23 | -4.243 | 18 |
| Coronavirus Pathogenesis Pathway | 8.85 | 3.333 | 40 |
| TP53 Regulates Metabolic Genes | 8.38 | -3.128 | 23 |
| Hematoma Resolution Signaling Pathway | 7.65 | -4.218 | 43 |
| Parkinson's Signaling Pathway | 7.2 | 6.332 | 48 |
| Regulation of mitotic cell cycle | 6.68 | -2.837 | 21 |
| Regulation of mRNA stability by proteins that bind AU-rich elements | 6.59 | -3.273 | 21 |

**EtOH+Capns1 KD vs EtOH –** **canonical pathways**

| Ingenuity Canonical Pathways | -log(p-value) | z-score | gene count |
| --- | --- | --- | --- |
| HEY1 Signaling Pathway | 8.38 | 1.508 | 11 |
| Molecular Mechanisms of Cancer | 4.72 | 1.5 | 17 |
| Cholesterol biosynthesis | 4.7 | -2 | 4 |
| CDX Gastrointestinal Cancer Signaling Pathway | 4.56 | -1.414 | 8 |
| Superpathway of Cholesterol Biosynthesis | 4.45 | -2 | 4 |
| G alpha (i) signalling events | 3.75 | 2.121 | 8 |
| Pulmonary Healing Signaling Pathway | 3.65 | 0.378 | 7 |
| Sheddase Signaling Pathway | 3.61 | 1.89 | 7 |
| S100 Family Signaling Pathway | 3.54 | 1.941 | 14 |
| Factors Promoting Cardiogenesis in Vertebrates | 3.5 | 0 | 6 |
| NCAM signaling for neurite out-growth | 3.24 | 1 | 4 |
| Role of Osteoblasts in Rheumatoid Arthritis Signaling Pathway | 3.18 | 1.134 | 7 |
| Role of Osteoclasts in Rheumatoid Arthritis Signaling Pathway | 3.16 | -1.342 | 8 |
| Zn Homeostasis Signaling Pathway | 3.04 | 0.5 | 16 |
| Cellular Effects of Sildenafil (Viagra) | 3.02 | 0.302 | 12 |
| RAR Activation | 2.88 | 0.333 | 9 |
| Cerebral Malformation Signaling Pathway | 2.83 | 1.342 | 5 |
| Endocannabinoid Cancer Inhibition Pathway | 2.7 | -2 | 5 |
| Activation of gene expression by SREBF (SREBP) | 2.68 | N/A | 3 |
| Tuberculosis Latent Signaling Pathway | 2.66 | 0 | 4 |
| BMP signaling pathway | 2.66 | 0 | 4 |
| Transcriptional Regulatory Network in Embryonic Stem Cells | 2.49 | -0.447 | 5 |
| Glutaminergic Receptor Signaling Pathway (Enhanced) | 2.43 | 1.89 | 7 |
| Wound Healing Signaling Pathway | 2.39 | 0 | 6 |
| Post-translational protein phosphorylation | 2.39 | -2 | 4 |
| Class A/1 (Rhodopsin-like receptors) | 2.38 | 0.378 | 7 |
| CREB Signaling in Neurons | 2.37 | 1 | 10 |
| Tumor Microenvironment Pathway | 2.32 | 0.447 | 5 |
| p38 MAPK Signaling | 2.17 | 0 | 4 |
| Regulation of Insulin-like Growth Factor (IGF) transport and uptake by IGFBPs | 2.17 | -2 | 4 |

**EtOH vs control - upstream regulators**

| Upstream Regulator | Molecule Type | z-score | p-value of overlap |
| --- | --- | --- | --- |
| PPARA | ligand-dependent nuclear receptor | 0.048 | 1.03E-46 |
| AHR | ligand-dependent nuclear receptor | -1.2 | 5.64E-42 |
| TGFB1 | growth factor | -3.227 | 1.43E-37 |
| STAT5B | transcription regulator | 3.412 | 3.74E-36 |
| NFE2L2 | transcription regulator | -3.797 | 1.98E-35 |
| RORA | ligand-dependent nuclear receptor | 0.217 | 7.86E-31 |
| PPARG | ligand-dependent nuclear receptor | 2.017 | 1.46E-30 |
| SREBF2 | transcription regulator | 4.575 | 9.86E-30 |
| AGT | growth factor | -1.223 | 2.47E-29 |
| MYC | transcription regulator | -2.963 | 1.67E-28 |
| NR1I3 | ligand-dependent nuclear receptor | -4.699 | 5.88E-28 |
| TP63 | transcription regulator | -2.303 | 6.09E-27 |
| TP53 | transcription regulator | -2.689 | 6.19E-26 |
| CEBPB | transcription regulator | -0.063 | 9.32E-25 |
| FOXO1 | transcription regulator | -1.287 | 2.72E-24 |
| ESR2 | ligand-dependent nuclear receptor | 0.094 | 5.24E-24 |
| NR3C1 | ligand-dependent nuclear receptor | -1.453 | 5.04E-23 |
| IGF1 | growth factor | -1.006 | 1.1E-22 |
| PPARD | ligand-dependent nuclear receptor | 1.489 | 1.86E-22 |
| RORC | ligand-dependent nuclear receptor | 1 | 2.52E-22 |
| FOXO3 | transcription regulator | -2.451 | 3.49E-22 |
| CEBPA | transcription regulator | -2.221 | 3.66E-22 |
| NFAT5 | transcription regulator | -0.236 | 1.44E-21 |
| HUWE1 | transcription regulator | 2.665 | 2.62E-21 |
| RXRA | ligand-dependent nuclear receptor | 1.819 | 1.84E-20 |
| HNF4A | transcription regulator | 1.997 | 4.04E-20 |
| HMG20A | transcription regulator | 1.417 | 5.81E-20 |
| SREBF1 | transcription regulator | 3.233 | 6.06E-20 |
| HTT | transcription regulator | -1.801 | 9.32E-20 |
| SIRT1 | transcription regulator | -2.819 | 1.09E-19 |

**Capns1 KD vs control – upstream regulators**

| Upstream Regulator | Molecule Type | z-score | p-value of overlap |
| --- | --- | --- | --- |
| MLXIPL | transcription regulator | -7.842 | 3.63E-49 |
| LARP1 | translation regulator | 7.547 | 1.9E-46 |
| SPEN | transcription regulator | -6.856 | 4.83E-42 |
| CTNNB1 | transcription regulator | 2.183 | 3.13E-30 |
| MYC | transcription regulator | -4.941 | 2.27E-26 |
| FMR1 | translation regulator | 6.378 | 5.4E-26 |
| YAP1 | transcription regulator | 1.031 | 3.12E-24 |
| HNF4A | transcription regulator | 0.987 | 1.21E-23 |
| EIF6 | translation regulator | 5.555 | 3.62E-21 |
| TEAD1 | transcription regulator | -4.922 | 9.01E-17 |
| MYCN | transcription regulator | -5.653 | 1.68E-16 |
| ZHX2 | transcription regulator | 4.591 | 1.27E-11 |
| HIF1A | transcription regulator | 1.229 | 1.28E-11 |
| PPARA | ligand-dependent nuclear receptor | -0.926 | 5.9E-11 |
| RB1 | transcription regulator | -4.816 | 7.9E-11 |
| CLPB | transcription regulator | 3.051 | 1.29E-08 |
| RXRA | ligand-dependent nuclear receptor | 1.359 | 0.000000235 |
| IGF2BP1 | translation regulator | 1.231 | 0.000000316 |
| COPS5 | transcription regulator | 3.06 | 0.00000042 |
| AGT | growth factor | 0.897 | 0.000000427 |
| HUWE1 | transcription regulator | 1.187 | 0.000000437 |
| STAT5B | transcription regulator | 1.523 | 0.000000467 |
| NR3C1 | ligand-dependent nuclear receptor | -1.008 | 0.00000075 |
| PPARGC1A | transcription regulator | -1.232 | 0.000000923 |
| NR1I3 | ligand-dependent nuclear receptor | -1.165 | 0.00000197 |
| TP53 | transcription regulator | -0.245 | 0.00000244 |
| NRF1 | transcription regulator | -3.074 | 0.00000279 |
| HTT | transcription regulator | -0.327 | 0.0000029 |
| PITX2 | transcription regulator | -2.855 | 0.00000297 |
| MYCL | transcription regulator | -2.926 | 0.00000324 |

**EtOH + Capns1 KD vs EtOH – upstream regulators**

| Upstream Regulator | Molecule Type | z-score | p-value of overlap |
| --- | --- | --- | --- |
| TGFB1 | growth factor | 0.831 | 1.1E-10 |
| NPM1 | transcription regulator | 0.333 | 0.000000214 |
| SMARCB1 | transcription regulator | -1.134 | 0.000000305 |
| ARID1A | transcription regulator | -2.401 | 0.00000729 |
| SREBF2 | transcription regulator | -2.607 | 0.0000108 |
| PGR | ligand-dependent nuclear receptor | -1.947 | 0.0000185 |
| SREBF1 | transcription regulator | -0.918 | 0.0000276 |
| EGF | growth factor | 1.52 | 0.0000292 |
| FOS | transcription regulator | 0.816 | 0.000043 |
| KMT2D | transcription regulator | -0.552 | 0.000068 |
| AHR | ligand-dependent nuclear receptor | 0.214 | 0.000177 |
| TP63 | transcription regulator | 2.09 | 0.000193 |
| AGT | growth factor | -0.777 | 0.00022 |
| TGFB3 | growth factor | 0.816 | 0.000234 |
| NCOA2 | transcription regulator | -0.816 | 0.000327 |
| ARID2 | transcription regulator | 0 | 0.000359 |
| BMP10 | growth factor | 0.447 | 0.000387 |
| ESR2 | ligand-dependent nuclear receptor | -1.667 | 0.000407 |
| GDF2 | growth factor | 0 | 0.000478 |
| SMAD3 | transcription regulator | -0.053 | 0.000491 |
| BMP2 | growth factor | 2.149 | 0.000613 |
| STAT5B | transcription regulator | 2.121 | 0.000649 |
| CTNNB1 | transcription regulator | -0.513 | 0.000727 |
| IGF1 | growth factor | 0.733 | 0.000802 |
| QKI | transcription regulator | -1.253 | 0.000854 |
| TARDBP | transcription regulator | -2 | 0.000913 |
| FOXO4 | transcription regulator | 1.06 | 0.000961 |
| NFKB2 | transcription regulator | -0.962 | 0.000999 |
| ESRRA | transcription regulator | -1 | 0.00139 |
| STAT3 | transcription regulator | -0.047 | 0.00165 |

**Figure legends**

**Supplementary Figure S1. Effect of ethanol exposure and Capns1 knockdown on indices of liver injury.**

To assess liver injury, we measured serum AST level, the expression of proinflammatory gene, *Tnfα*, and immune cell markers, *Ly6g* (neutrophils), *Cd68* (inflammatory macrophages), and *F4/80* (Kupffer cells and monocytes) in addition to body weight and liver weight. AAV, adeno-associated virus; PF, pair-fed; EtOH, ethanol-fed; AST, aspartate aminotransferase.

**Supplementary Figure S2. Effect of ethanol exposure and Capns1 knockdown on lipid metabolism.**

Panel A: The expression of key lipid metabolism genes was determined. These included genes involved in fatty acid metabolism (*Fasn*) and cholesterol homeostasis (*Lcat*). Panel B: The protein levels of mediators of cholesterol metabolism were determined. Specifically, the expression of ABCA1, ABCG1 and VCP was determined. AAV, adeno-associated virus; PF, pair-fed; EtOH, ethanol-fed.

**Supplementary Figure S3. Canonical pathways and upstream regulators identified by Ingenuity Pathway Analysis (IPA) in each group comparison from the RNA-seq dataset.** (A) EtOH vs Control, (B) Capns1KD vs Control, and (C) EtOH+Capns1KD vs EtOH. Pathways are ranked by –log10 p-value, and z-scores are shown where available. Positive z-scores represent predicted activation, whereas negative z-scores represent predicted inhibition. NA indicates no z-score available. IPA, Ingenuity Pathway Analysis; KD, knockdown.

**Supplementary Table 4. Enriched canonical pathways and upstream regulators (IPA analysis).**

Canonical pathways and upstream regulators identified by Ingenuity Pathway Analysis (IPA) in each group comparison from the RNA-seq dataset. Pathways are ranked by –log10 p-value. Predicted activation or inhibition is indicated by z-score, with positive values representing activation and negative values representing inhibition. NA indicates that a z-score could not be calculated. Gene symbols are displayed in uppercase (e.g., SREBF2, ABCA1) based on IPA output, while gene symbols in the main text follow mouse nomenclature (e.g., *Srebf2, Abca1*).
